# Leptin expression affects metabolic rate in zebrafish embryos (*D. rerio*)

**DOI:** 10.3389/fphys.2013.00160

**Published:** 2013-07-01

**Authors:** Mark R. Dalman, Qin Liu, Mason D. King, Brian Bagatto, Richard L. Londraville

**Affiliations:** Integrated Bioscience Program, Department of Biology, University of AkronAkron, OH, USA

**Keywords:** leptin-A, zebrafish, rescue, metabolic rate, oxygen consumption, carbon dioxide microplate assay

## Abstract

We used antisense morpholino oligonucleotide technology to knockdown leptin-(A) gene expression in developing zebrafish embryos and measured its effects on metabolic rate and cardiovascular function. Using two indicators of metabolic rate, oxygen consumption was significantly lower in leptin morphants early in development [<48 hours post-fertilization (hpf)], while acid production was significantly lower in morphants later in development (>48 hpf). Oxygen utilization rates in <48 hpf embryos and acid production in 72 hpf embryos could be rescued to that of wildtype embryos by recombinant leptin coinjected with antisense morpholino. Leptin is established to influence metabolic rate in mammals, and these data suggest leptin signaling also influences metabolic rate in fishes.

## Introduction

Leptin is a 16 kD cytokine hormone that was discovered nearly two decades ago; mice with a homozygous (*ob^−^/ob^−^*) mutation have significant hyperphagia and increased adipose storage (Zhang et al., 1994). Leptin has been cloned across several vertebrate species, including mammals, amphibians, reptiles, and fish (Kurokawa et al., 2005; Crespi and Denver, 2006; Boorse and Libbon, 2010). In mammals, leptin expression is highest in adipose tissue whereas among fishes, liver is the major expressing tissue (Gorissen et al., 2009; Liu et al., 2010). Mammalian leptin binds to JAK-STAT receptors in the hypothalamus, which activate anorexigenic pathways; leptin signaling is also present in fishes (Londraville and Duvall, 2002; Murashita et al., 2008). Non-mammal leptins do not share conserved primary structure with mammalian leptins (10–30%); yet tertiary structure and some aspects of leptin function appear to be well conserved across vertebrates (Johnson et al., 2000; Londraville and Niewiarowski, 2010). In mammals, leptin injections result in a dose-dependent reduction in food intake and increased lipolysis, metabolic rate, and thermogenesis (Makimura et al., 2001). In goldfish, intracerebroventricular (ICV) injection of murine leptin reduces food intake (Volkoff et al., 2003; de Pedro et al., 2006). Intraperitoneal (IP) injections of fish leptin reduce appetite (short term) in rainbow trout (Murashita et al., 2008), though IP delivery does not affect appetite in Coho salmon, catfish, or green sunfish (Baker et al., 2000; Silverstein and Plisetskaya, 2000; Londraville and Duvall, 2002). These contradictory results are likely confounded by leptin source (native or heterologous), method of delivery (IP or ICV), dosage, and sampling interval. Therefore, it is difficult to assess which aspects of leptin function are conserved from fish to mammals.

The availability of mutants without leptin signaling has been invaluable to advancing leptin biology. *Ob^−^/ob^−^* mice are signaling as if nutrient starved, and show similar dysfunction to fasted animals such as decreased thermogenesis, hypometabolism, infertility, and hyperphagia. All of these abnormalities can be rescued via endogenous leptin injections, however injections in fed wildtype mice do not significantly increase metabolic rate nor decrease food intake (Mistry et al., 1997; Doring et al., 1998). Moreover, diet induced obesity (DIO) and *ob^−^/ob^−^* mice show similar but not identical gains in body weight (de Pedro et al., 2006). These studies suggest that manipulating leptin in an animal that does not express the hormone is fundamentally different than manipulating leptin in one that does. To date, leptin investigations in non-mammals have been hampered by the lack of leptin-null mutants.

We now know that leptin's effects go beyond appetite, with influence on immune function, reproduction, bone, and metabolism. To date, the effects of acute leptin injection are unresolved with variable responses depending on development age and time of the injection (Fruhbeck, 1999; Mistry et al., 1999; Bagatto and Burggren, 2006). The current paradigm is that leptin increases oxygen consumption in mammals and reptiles while also increasing heart rate, mean arterial pressure, and sympathetic tone [through the CNS and peripheral tissues; (Winnicki et al., 2001; Carlyle et al., 2002; Chu et al., 2010)]. Zebrafish (*D. rerio*) embryos provide a robust model to visualize cardiac output noninvasively due to their transparency. Additionally, the availability of molecular tools makes it a useful model to test leptin function in early vertebrates (Barrionuevo and Burggren, 1999; Liu et al., 2010, 2012).

We have developed and characterized both zebrafish leptin and leptin receptor-knockdown models (Liu et al., 2010, 2012). We used antisense morpholino oligonucleotides against zebrafish leptin-A, resulting in embryos with dramatically reduced leptin expression and effects on the development of heart, eye, inner ear, and notochord (Liu et al., 2012). We established that the leptin A-knockdown is specific through a series of controls, including (1) leptin mRNA knockdown reduces zebrafish leptin expression (2) the morphant phenotype is produced with either several morpholinos against leptin or the leptin receptor, and control morpholinos produce no apparent phenotype (3) the morphant phenotype is rescued with recombinant leptin (Liu et al., 2012). Here we present effects of that knockdown on metabolic rate and cardiac function. Leptin's effects on metabolic rate were measured directly by oxygen microprobe and indirectly via a pH sensitive dye for acid production. Cardiac output was analyzed using a high-speed camera and image software. In general, leptin A knockdown reduced metabolic rate and was rescued by recombinant leptin, although the effect was dependent on developmental age and assay method.

## Material and methods

### Animals

Wild-type adult zebrafish (Aquatic Tropicals, Bonita Springs, FL), *Danio rerio*, were maintained and bred at 28.5°C with a light cycle of 14L:10D, according to The Zebrafish Book (Westerfield, 1994). Immediately after fertilization, zebrafish embryos were transferred to fish tank water with fungicide (0.05% methylene blue) and allowed to develop. Ages of the embryos or larvae are given as hours post-fertilization (hpf). All animal-related procedures were approved by the University of Akron Institutional Animal Care and Use Committee (IACUC).

### Morpholino design and rescue

Morpholino antisense oligonucleotides were designed and manufactured by Gene Tools (Philomath, OR) and reconstituted in Daneau buffer [58 mM NaCl, 0.7 mM KCl, 0.4 mM MgSO_4_, 0.6 mM Ca(NO_3_)_2_, 5.0 mM HEPES pH 7.6]. A leptin-A translation blocking morpholino (lepMO: 5′-TTG AGC GGA GAG CTG GAA AAC GCA T -3′), a zebrafish leptin- receptor translation blocking morpholino (lepRMO: 5′- TCA AGA CAG ACA TCA TTT CAC TTG C -3′), and a control MO with five-mismatched nucleotides to LepMO (5-misMO: 5′- TTG AcC GcA GAc CTG cAA AAg GCA T -3′) were used in this study (Liu et al., 2012). These morpholinos have been previously validated for both leptin and leptin receptor (Liu et al., 2010, 2012). Embryos at the 1–8 cell stage were injected with 2 nl of morpholino at a concentration of 0.4 mM using a Narishige MI300 microinjector. Subsamples of embryos at each age were used for each experiment at each respective age. Zebrafish leptin A protein (recombinant, GenScript) was co-injected with the leptin morpholino to “rescue” the morphant phenotype. A leptin protein stock solution (30 μM in 50 mM Tris, pH 8.0, >90% pure) was co-injected with the leptin-A morpholino and the embryos allowed to develop as above (Liu et al., 2012).

### Oxygen consumption

Dechorionated zebrafish embryos at desired ages (10/trial) were placed into 5 ml vials and allowed to acclimate for 10 min at 28.5°C. Each measurement was repeated with previously untested fish 2–9X, with 10 embryos/measurement. Three initial measurements were conducted using an oxygen microelectrode connected to Power Lab (ADInstruments, Colorado Springs, CO) with LabChart software (ADInstruments, Colorado Springs, CO). After initial readings, vessels were sealed with parafilm and incubated for an hour, after which, three oxygen consumption measurements were taken. Mean consumption rates were displayed as nmol of O_2_ per individual hour. Parafilm is slightly permeable to gas exchange but the small surface area of the vial potentially exposed to atmospheric gases and the small duration of the measurement (1 h) had no significant influence on oxygen consumption measurements.

### Colorimetric whole-animal acid production assay

This assay was adapted from Makky et al. (2008). One dechorionated embryo was placed in a series of three washes of phenol red (0.02% w/v) assay medium (consisting of RO water supplemented with Instant Ocean to a conductivity of ~350 μS at 28.5°C) adjusted to pH 8.0 with sodium bicarbonate solution. Once rinsed and acclimated, 100 μl of assay medium (with embryo) was transferred to one well of a sterile polystyrene 96-well microplate (Evergreen Scientific, Los Angeles, CA). Coverslip mineral oil (100 μl) was overlaid to diminish diffusion of environmental oxygen into each well. Absorbance was measured at 570 nm over 1 h at 28.5°C and 10-s intervals using a spectrophotometer (Spectramax 384 Molecular Devices, Sunnyvale, CA). Cumulative acid production was then derived from absorbance using a previously described equation (Makky et al., 2008). Sample size was ≥4 for each measurement.

### Heart rate, stroke volume, and cardiac output

Video of the beating heart was captured for each individual embryo using a high-speed digital camera (Red Lake MASD, San Diego, CA) on an inverted microscope with a temperature-controlled stage. Early larva rested on the chamber bottom and typically did not move, however after swim bladder inflation, larval movement required light anesthesia with 0.002% tricaine (MS-222). This low concentration of MS-222 does not affect heart rate compared to free swimming larvae (Moore et al., 2006). Heart rate was measured by recording time elapsed over 15 heartbeats and extrapolating to beats per minute (3x/larvae). Stroke volume was estimated from end systole and diastole area recorded in a single frame for each during the cardiac cycle (Moore et al., 2006).

### Statistics

Oxygen consumption, microplate assay and cardiovascular data were analyzed using a two-way analysis of variance (ANOVA) at a *p* = 0.05 using standard linear models analysis (SAS Institute, Cary, NC) with developmental age and treatment (wildtype, leptinMO, and rescue) as factors. Data were distributed normally. A Tukey's multiple comparison procedure was performed to assess for specific pair-wise comparisons *post-hoc*.

## Results

### Oxygen consumption

**Figure 1:**
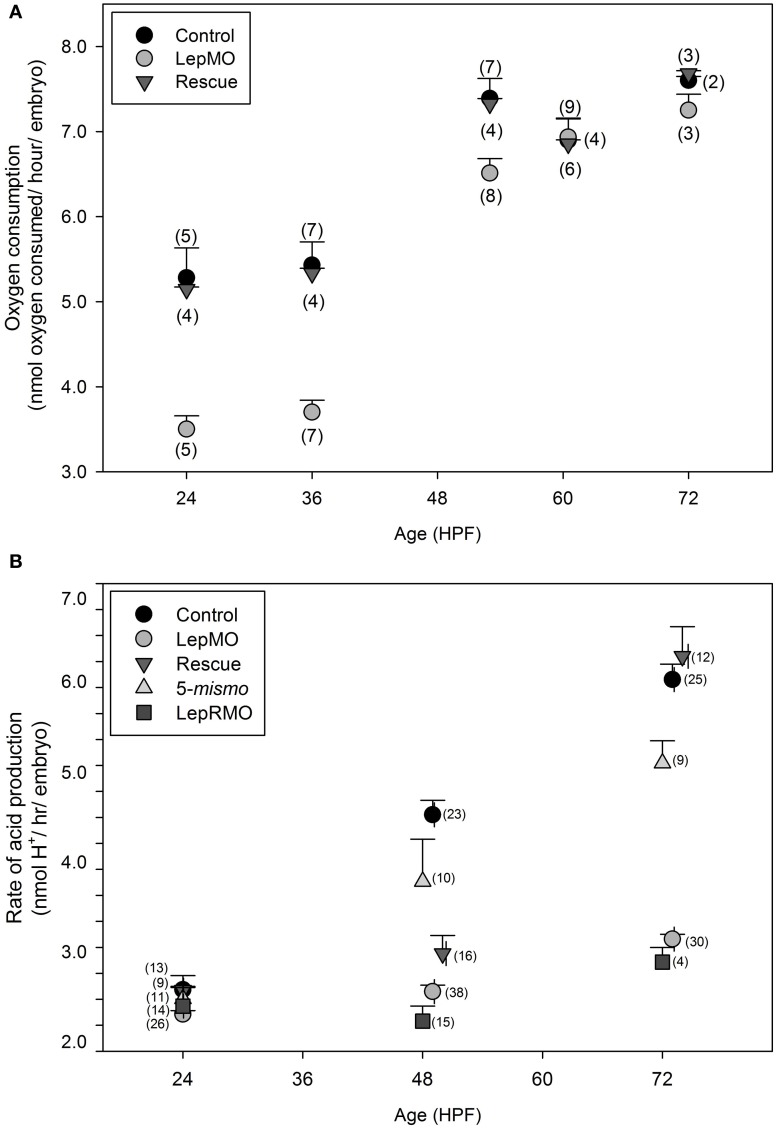
**(A)** Oxygen consumption per individual for wildtype (control), morphants (LepMO), and rescue (morpholino + recombinant zebrafish leptin) embryos. All time points are significantly different (*p* < 0.05) between morphants and control and morphants and rescue, with the exception of 60 hpf (*N* = measurements/age, 10 individuals/measurement). **(B)** Cumulative acid production measured per embryo/hour by change in absorbance @570 nm (*N* = individual fish). All data are mean ± SE. Control, wildtype embryo; LepMO, leptin morpholino injected zebrafish embryo; Rescue, Co-injected LepMO morpholino with recombinant zebrafish leptin; 5-mismo, control morpholino with mismatch basepairing at 5 sites; LepRMO, leptin receptor morpholino. All fish are aged matched. No significant differences among treatments at 24 hpf; Control and 5-mismo are significantly higher than other treatments at 48–50 hpf, and LepMO and LepRMO are significantly lower than other treatments at 72–76 hpf. *p* < 0.05.

Oxygen consumption rate of leptin morphants was significantly decreased compared to both control and rescue embryos at all time points (*p* < 0.01) except for 60 hpf (Figure 1A, Table 1A). Each datum comprised an average of 10 embryos of a standardized mass for that developmental age as previously described by Barrionuevo and Burggren (1999) and Bang et al. (2004).

**Table 1:** **Sources of variation in metabolic rate**.

**Source**	**dF**	**MS**	**F**	**Pr > F**
**(A) TWO-WAY ANOVA FOR OXYGEN UTILIZATION**				
Developmental age	4	25.1	113.8	<0.001
Treatment	2	6.6	29.8	<0.001
Age × treatment	8	1.2	5.3	<0.001
Error	63	0.2		
**(B) TWO-WAY ANOVA FOR ACID PRODUCTION**				
Developmental age	2	208.7	149.5	<0.001
Treatment	4	102.1	73.0	<0.001
Age × treatment	8	30.15	21.6	<0.001
Error	240	1.4		

ANOVA was run using developmental age, treatment, and age by treatment interaction as the modeled sources of variation.

### Colorimetric aggregate acid production assay

All assay plate experiments were run in parallel such that a control well (assay medium only), a wildtype embryo, and a leptin-MO injected embryo (age matched) were placed in adjacent wells and each embryo only assayed once. The rate of total acid production was interpreted as the linear regression of changes in optical density at 570 nm over time. Blank wells showed no significant drift throughout the 1-h measuring window. Data were adjusted for morphological age but not for size or mass as previously described (Liu et al., 2010, 2012). Although morphant embryos are slightly smaller than wildtype (7.5% difference in total length), we compared morphants to wildtype and rescues using the same standardized mass (Bang et al., 2004), because any decrease in length is compensated by an increase in yolk (Liu et al., 2012). Control (wildtype) and morphant embryos absorbance values were blanked at time zero. In aggregate, there were significant effects of treatment, age, and an interaction between treatment and age (Figure 1B, Table 1B, *p* < 0.001), although these effects were driven by differences at 48 and 72 hpf, not 24 hpf (Figure 1B). The morpholino's efficacy is reduced beginning ~4 dpf (Liu et al., 2010, 2012), therefore morphants were not assayed at 96 hpf. Leptin morphants rescued with recombinant leptin had metabolic rates equivalent to wildtype at 24 and 72 hpf, but not 48 hpf (Figure 1B).

### Cardiac output

**Figure 2:**
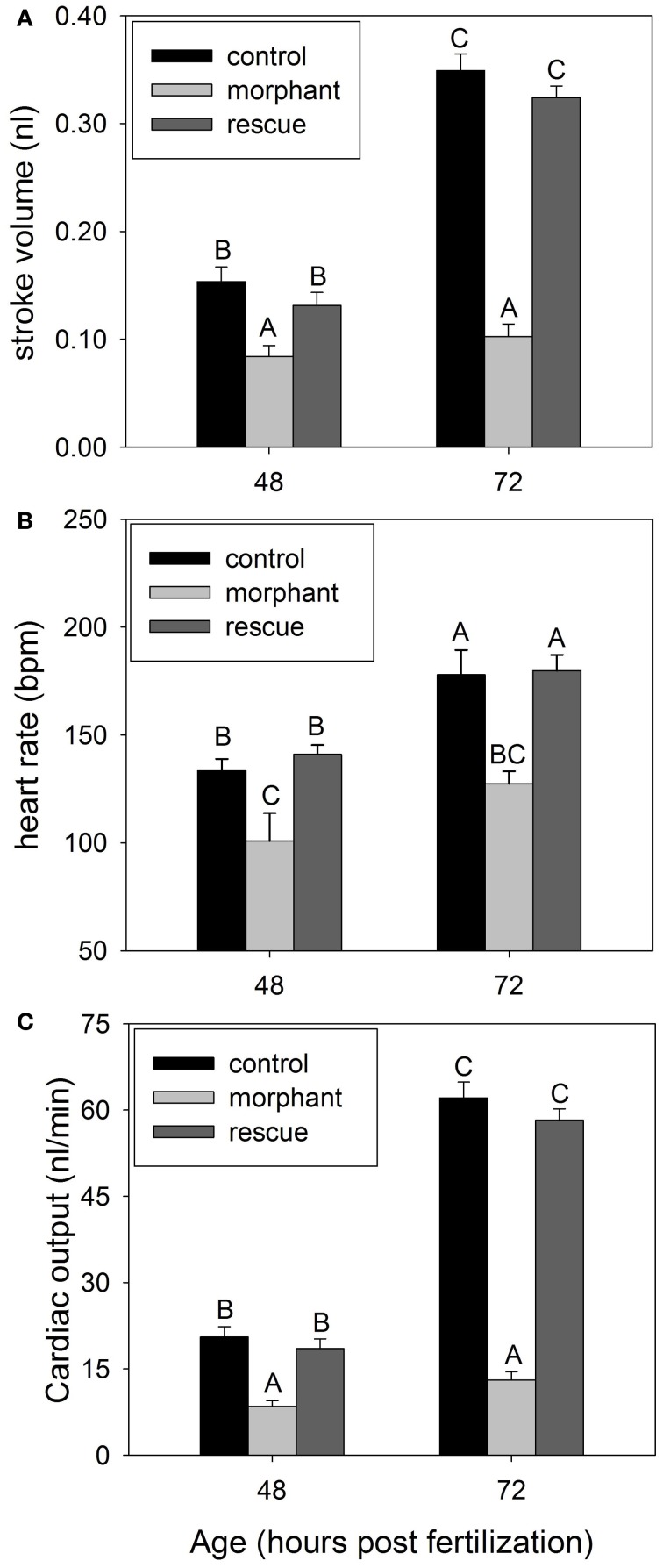
**(A)** Average stroke volume for wildtype (control), leptin morphants (morphant), and rescue (leptin morphant + recombinant zebrafish leptin) at two developmental timepoints. **(B)** Embryonic heart rate for wildtype (control), leptin morphants (morphant), and rescue (morphant + recombinant zebrafish leptin) at two developmental timepoints. **(C)** Average cardiac output for wildtype (control), leptin morphants (morphant), and rescue (leptin morphant + recombinant zebrafish leptin) embryos. All data are mean ± SE. (*N* = 5 for **A, B**, *N* = 7 for **C**). Bars that do not share letters are significantly different (*p* < 0.05).

There is inherently high variability among individuals in embryonic development at 24 hpf (Westerfield, 1994; Bang et al., 2004), therefore ventricle area was measured at 48 and 72 hpf only. Heart rate was lower in morphants with a pronounced hesitation between each heartbeat. Heart rate generally increased between 48 and 72 hpf, except for morphants (Figure 2). Stroke volume and cardiac output increase dramatically between 48 and 72 hpf for wildtype embryos, but not morphants. Coinjection of recombinant leptin A returns all cardiac variables to that of wildtype (Figure 2).

## Discussion

Leptin's effect on metabolic rate was one of the first functions described for the hormone, but its effects on fish metabolic rate were undescribed. Here we test the effects of leptin A knockdown on metabolic rate for the first time in a non-mammal. In general, the results indicate that leptin knockdown reduces metabolic rate and cardiac output, and that these effects can be rescued by recombinant leptin A. These effects differ depending on developmental age and assay.

The end metabolite of both aerobic and anaerobic metabolism is acid. CO_2_ is respired during aerobic metabolism and when hydrated produces carbonic acid, whereas lactic acid is the end metabolite during anaerobic metabolism. These products of cumulative metabolism are commonly used as indicators of metabolic rate (Hu et al., 2000; O'Connor et al., 2000; Stackley et al., 2011), however the validity of oxygen consumption methodologies are debated for aquatic organisms, especially those observed under a microscope (Makky et al., 2008). Unstirred boundary layers, diffusion, tissue density, probe precision, and clutch effects all can influence oxygen consumption measurements at the microscopic level (Feder and Pinder, 1998; Bang et al., 2004; Moore et al., 2006; Makky et al., 2008). However, the field has established that absolute metabolic rate increases over developmental time, mass-specific metabolic rate decreases, and both neuroendocrine and autonomic nervous systems can influence metabolic rate (Zhang and Wang, 2006; Fraisl et al., 2009).

We calculate oxygen consumption at 3.5–8.0 nmol O_2_/h (Figure 1A) during development, which corroborates previously documented fish embryo data [4.54–8.29 nmol O_2_/h (Bang et al., 2004)]. Lower published values (2 nmol O_2_) may be explained by differences in methodology (Barrionuevo and Burggren, 1999). Our data are reported per embryo rather than per gram as approximately 60% of an embryo's surface area is yolk sac that is facilitating only 33% of the overall oxygen uptake through diffusion (Wells and Pinder, 1996). Therefore, relatively metabolically inactive yolk may unduly influence metabolic rate calculations on a per gram basis (Breslow et al., 1999). Finally, as further verification of this assay, we document a slowing of metabolic rate >48 hpf. This is consistent with Kimmel et al. (1995), who demonstrated that the rate of change for the head-trunk angle dramatically slows during this time period, as does cardiac output in control zebrafish (Bagatto et al., 2006).

We used an aggregate acid production colorimetric assay to expand our dataset due to its higher precision (individual measurements vs. groups of 10) and greater throughput. Oxygen consumption and acid production have been widely used and validated for multiple dyes and microplate assays (Rowell, 1995; Campbell et al., 2003; Nieman et al., 2003; O'Mahony et al., 2005; Makky et al., 2008; Stackley et al., 2011). Total aggregate acid for developing zebrafish embryos increases over developmental time (Makky et al., 2008; Stackley et al., 2011). Our data fall within 2.10–6.95 nanomoles of H^+^ produced per hour per embryo, and are consistent with Makky et al.'s original study (Makky et al., 2008). Leptin morphants had significantly reduced rates of aggregate acid production (Figure 1B, *p* < 0.001). To minimize effects of decreasing pH as the assay proceeds, all data were collected over 1 h. Further, pH change (aggregate acid production) fit to a linear regression with *r*^2^ > 0.95 (data not shown) suggesting assay conditions did not change significantly during the assay, or if they did, they did not affect metabolic rate.

We used two measures of metabolic rate to test the effects of leptin knockdown; both are indirect measures of organismal metabolic activity. Although we can verify that our data are consistent with previously published control zebrafish data for each technique, and we can demonstrate an effect of knockdown and rescue with each assay, the response of each developmental age is not the same between assays. For both assays, there is a significant interaction between developmental age and treatment. For oxygen utilization, the effect of leptin knockdown decreases with time (Figure 1A), and for acid production, the effect increases with time (Figure 1B). Clearly, even though both of these assays are routinely used to measure metabolic rate (Nieman et al., 2003; Makky et al., 2008; Stackley et al., 2011), leptin expression affects each in distinct ways. Leptin knockdown decreasing its effect on oxygen utilization over time is consistent with the activity profile of morpholinos (losing their acitivity over the course of 4 days). We speculate that leptin knockdown canalizes some event early in development that accounts for more and more of acid production as development proceeds [e.g., proton leak, (Stackley et al., 2011)]. Going forward, each assay has advantages. Oxygen utilization certainly has the weight of decades of research supporting its use, but the sensitivity and “ease of use” of oxygen electrodes make it impractical for individual embryos. The colorimetric assay has the advantage of precision, throughput, and ease of use. We recognize that the colorimetric assay does not distinguish between anaerobic or aerobic metabolism, however the relative contribution of lactic acid to hydrated CO_2_ for developing zebrafish embryos is minimal, suggesting most of the aggregate acid measured is from the hydration of CO_2_ (Stackley et al., 2011). We assert that the colorimetric assay is precise and robust in determining relative, if not absolute differences in metabolic rate among treatments.

Cardiac output is tightly coupled with metabolism and the cardiovascular development of the zebrafish heart has been widely documented along with the ontogeny of its control (Hu et al., 2000; Bagatto, 2001). Our data demonstrate that leptin knockdown in developing embryos significantly decreases heart rate and stroke volume, resulting in significant decreases in cardiac output (Figure 2). *Ob^−^/ob^−^* mice have reduced arterial pressure and heart rate compared to wildtype (Mark et al., 1999). The mechanism by which leptin exerts its action on cardiac output is proposed to be increased sympathetic tone, causing changes in heart rate, vascular tone, and the contractile properties of ventricular myocytes in mammals (Ozata et al., 1999; Nickola et al., 2000). Morphant zebrafish rescued with recombinant leptin returns heart rate to that of wildtype embryos, suggesting that leptin may exert its effects on metabolic rate partially through modulating heart rate (Figure 2).

We sought to determine if leptin A influences metabolic rate in zebrafish, similar to how leptin affects metabolic rate in mammals. Although the assays we used do not agree with the magnitude or timing of the effect, we assert that the data supporting a role of zebrafish leptin A in modulating metabolic rate are robust. In addition, we demonstrate that zebrafish cardiac performance is affected by leptin expression. By comparing how fish and mammal leptins are similar and divergent in structure and function, we hope to gain insight into the evolution of this hormone.

## Author contributions

Mark R. Dalman was involved in all aspects of the study. Qin Liu, Mason D. King, and Brian Bagatto were instrumental in data acquisition. Richard L. Londraville was involved in design and preparation of manuscript. Manuscript was reviewed by Mark R. Dalman, Qin Liu, Brian Bagatto, and Richard L. Londraville.

### Conflict of interest statement

The authors declare that the research was conducted in the absence of any commercial or financial relationships that could be construed as a potential conflict of interest.
